# A137 TRANSANAL ENDOSCOPIC COOPERATIVE SURGERY (TECS): COMBINED APPROACH FOR A LARGE POST-TRANSANAL RESECTION RECURRENCE. A CASE REPORT

**DOI:** 10.1093/jcag/gwac036.137

**Published:** 2023-03-07

**Authors:** C Pattni, T Chesney, J Mosko

**Affiliations:** 1 Division of Gastroenterology and Hepatology, University of Toronto; 2 Division of General Surgery, Unity Health; 3 Division of General Surgery, University of Toronto; 4 Division of Gastroenterology, St. Michael's Hospital, Toronto, Canada

## Abstract

**Background:**

Advancements in endoscopy and minimally invasive surgery have led to the development of organ-preserving resection techniques to manage rectal lesions. According to JGES, ESGE, and AGA, endoscopic mucosal dissection (ESD) is typically recommended for Kudo V/JNET 2b pit pattern, LST-NG/Paris Is/IIc/IIa+Is morphology, presence of submucosal fibrosis, and local residual/recurrent adenomas. ESD is not without its limitations including depth of resection, technical difficulty and time. In some instances, a transanal minimally invasive surgery (TAMIS) approach can overcome some of these limitations while also avoiding the morbidity of proctectomy. The ESD-TAMIS approach has been described by two groups in the USA and Germany demonstrating initial safety and feasibility for management of distal rectal adenomas.

**Purpose:**

We present the first Canadian case to demonstrate the use of a combined ESD-TAMIS for the management of a large post-TAMIS recurrent rectal adenoma, involving the dentate line, not amenable to endoscopic resection or TAMIS alone.

**Method:**

N/A

**Result(s):**

A healthy 78-yr-old woman was referred for consideration of ESD of a large recurrent low rectal adenomatous lesion which had undergone multiple prior endoscopic mucosal and TAMIS resections. Pathology from prior excisions never showed high grade dysplasia (HGD) or intramucosal cancer. The lesion was 4-5cm long and occupied 90% of the circumference of the lumen including the dentate line. It had Paris IIa+Is+IIb components with submucosal fibrosis. The pit pattern was JNET 2a/2b (Fig 1). Given the size and dramatic submucosal scarring, ESD was deemed extremely challenging, time consuming and would likely involve a full thickness resection. Similarly, given that the lesion involved the dentate line and the borders were difficult to delineate, a TAMIS would not be feasible. In collaboration with our surgical colleagues, we proceeded with the ESD-TAMIS. The borders of the lesion were first demarcated and marked endoscopically. Using standard ESD technique, a mucosal incision was performed on the anal side of the lesion, followed by submucosal dissection until sufficient space was created for the TAMIS platform. Then, using TAMIS technique, a full thickness resection was performed. The lesion was successfully removed en-bloc and pinned for pathology. The large size of the defect and proximity to the dentate line prevented surgical closure. Post-op course was complicated by fever successfully managed with antibiotics. Overall, the procedure was well tolerated. The final pathology revealed a tubulovillous adenoma with HGD and extensive submucosal fibrosis.

**Image:**

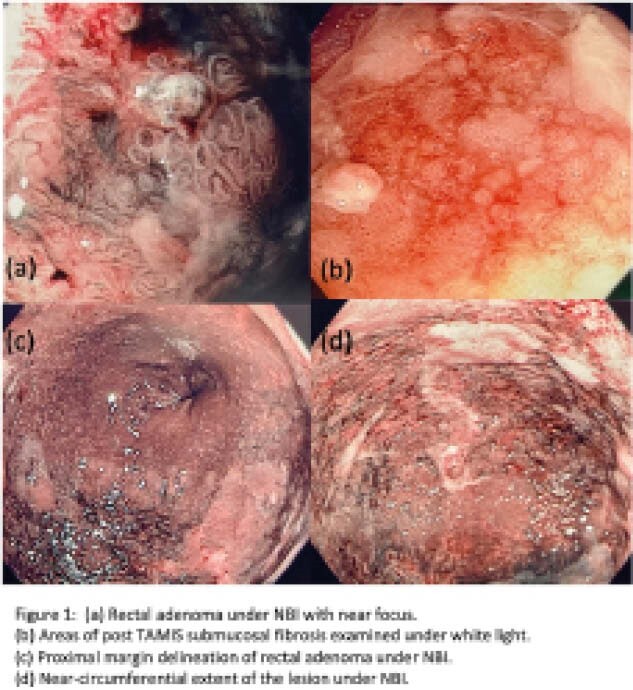

**Conclusion(s):**

A transanal endoscopic cooperative surgery combining ESD and TAMIS was safe and effective, combining the advantages of each procedural technique, in providing curative en-bloc resection of a recurrent large near-circumferential low rectal tubulovillous adenoma with HGD involving the dentate line. Here, we present the first reported Canadian case using this novel technique.

**Please acknowledge all funding agencies by checking the applicable boxes below:**

None

**Disclosure of Interest:**

None Declared

